# Low Prevalence of Rotavirus and High Prevalence of Norovirus in Hospital and Community Wastewater after Introduction of Rotavirus Vaccine in Nicaragua

**DOI:** 10.1371/journal.pone.0025962

**Published:** 2011-10-07

**Authors:** Filemón Bucardo, Per-Eric Lindgren, Lennart Svensson, Johan Nordgren

**Affiliations:** 1 Department of Microbiology, University of León, León, Nicaragua; 2 Division of Molecular Virology, Department of Clinical and Experimental Medicine, Linköping University, Linköping, Sweden; 3 Division of Medical Microbiology, Department of Clinical and Experimental Medicine, Linköping University, Linköping, Sweden; Tulane University, United States of America

## Abstract

Rotavirus (RV) and norovirus (NoV) are major causes of pediatric diarrhea and are altogether associated with approximately 800,000 deaths in young children every year. In Nicaragua, national RV vaccination program using the pentavalent RV5 vaccine from Merck was implemented in October 2006. To determine whether RV vaccination decreased the overall number of RV infections, we investigated the occurrence of RV and NoV in wastewater in the city of León from July 2007 to July 2008 and compared these data with pre-vaccination data. The major finding was the low prevalence of RV compared to NoV in all sampling points (11% vs 44%, p<0.05), and that RV concentration was lower as compared to NoV. RV was observed mainly during the rainy season (July–September), and the majority of all RV detected (6/9) belonged to subgroup (SG) I. The partial VP7-gene obtained from one RV positive sample was similar (99% nt identity) to a G6 VP7-gene of bovine origin and similar to the corresponding gene of the vaccine strain (98%). Furthermore RV G-types 2 and 4 were found in the incoming wastewater. NoV strains were detected throughout the year, of which a majority (20/21) were of genotype GII.4. We conclude that the introduction of RV vaccination reduced the transmission of RV in the community in Nicaragua. However, the burden of diarrhea in the country remains high, and the high prevalence of NoVs in hospital and municipal wastewater is noteworthy. This study highlights the need for further assessment of NoV following RV vaccine introduction.

## Introduction

Rotavirus (RV) causes approximately 111 million episodes of diarrhea and is associated with 527,000 deaths in young children every year, predominantly from developing countries. Norovirus (NoV) causes 900,000 clinical visits in industrialized countries and up to 200,000 deaths in children from developing countries [1], [2], [3]. Rotavirus is classified in serogroups (A–G), and based on molecular analysis of VP6 encoding gene, group A RV are divided in subgroups (SGI and SGII) [4], [5]. After safety and efficacy data evaluation of the two licensed RV vaccines (Rotarix and RotaTeq) the World Health Organization (WHO) strongly recommended the inclusion of RV vaccines in the Expanded Program of Immunization (EPI) of vulnerable countries [6]. From October 2006, the RotaTeq vaccine from Merck (RV5) is administered to all Nicaraguan children that are age-eligible.

RV and NoV typically infect the cell lining of the gastrointestinal tract and are discharged in very large numbers in the feces of symptomatic and asymptomatic infected persons (>1×10^8^ virus particles per grams of feces) [7], [8], [9]. Several reports indicate that both viruses are frequently found in water contaminated with human feces and detection rates follow a temporal pattern [10], [11], [12], [13], [14]. Viral concentration of RV and NoV can range between 1×10^4^ and 2×10^7^ genome equivalents per liter (g.e l^−1^) of raw wastewater [12], [15], [16].

By measuring enteric viral levels and determining the corresponding genotypes in wastewater samples, it is possible to indirectly monitor the presence of these enteric agents in the community. Previous studies have found a correlation between enteric viruses in wastewater and viruses causing disease in the community [14], [17], [18]. Furthermore, these types of investigations enable a more complete view of virus diversity at community level by also detecting viral strains that give rise to mild or asymptomatic infections, which are generally not observed at the clinical level [12], [17].

In this study, our objective was to determine whether the introduction of the RV5 vaccine in Nicaragua had reduced the circulation of RV in the community, and if shedding of RV vaccine strains had occurred into wastewater. Furthermore, we wanted to determine the temporal circulation of NoV and RV during one-year in Nicaragua after RV vaccination. This was carried out by performing a follow-up study of these viruses in sewage from the local hospital and the main wastewater treatment plant (WWTP) in the city of León after introduction of the pentavalent RV vaccine (RV5) in the EPI in Nicaragua.

In this study RV and NoV prevalence in wastewater was investigated in a country of Central America which has implemented RV vaccination in its national immunization schedule. This is also the first study to characterize enteric viruses in wastewater in Central America.

## Materials and Methods

### Site description

This study was carried out in the city of León, located close to the pacific cost of Nicaragua with an estimated population of 200,000 inhabitants. The climate is tropical; the rain season starts in June and lasts until November, when the dry season starts. Nicaragua was the first GAVI-eligible country to introduce rotavirus vaccination in October 2006. The Merck RV5 vaccine is provided to all Nicaraguan children that are age-eligible [19].

### Description of the wastewater treatment plant (WWTP)

The wastewater treatment plant was designed for clarification and sedimentation of wastewater. In the first step solids are removed by filtration. In the second step, large basins hold wastewater for a period of time, allowing soil sediments or other materials suspended in water to settle at the bottom. The last step is to discard the clarified water into the local river (Rio Chiquito). This WWTP receives wastewater from approximately 70% of the households of León, neighborhoods along the river including the historical center and the “Hospital Escuela Oscar Danilo Rosales” (HEODRA). During the dry and rainy seasons approximately 235 l/s and 336 l/s pass through the WWTP, respectively (personal communication, Lic. Maria José Diaz). The clarified water discharged by the WWTP eventually reaches the Pacific Ocean through an estuary located at the pacific coast (Las Peñitas) of Nicaragua.

### Wastewater samples

From July 2007 to July 2008 a total of 84 wastewater samples were collected from the inflow and outflow of the WWTP as well as from the wastewater drainer of the hospital (HEODRA). All necessary permits were obtained for the described field studies by the Director of HEODRA and the responsible for water affairs (ENACAL) in León. Weekly sampling was carried out every Thursday morning, from July to December 2007, to cover the rainy season, which coincides with the yearly epidemic peak of diarrhea after the RV vaccine introduction [20]. Monthly sampling (n = 1) was then carried out the second Thursday of each month from February until July 2008. No samples were collected in January 2008 due to logistic constrains. A grab sample was collected from the sewage drainer of the hospital, where wastewater can be collected before reaching the public system, between 7 and 8 AM (high flow). Then, between 9 and 10 AM, the same day 2 grab samples were collected from the inflow (before the mechanical filter) and outflow of the WWTP, respectively. At each site, 50 ml of sewage water was collected in sterile BD Falcon™ conical tubes (Becton, Dickinson and Company), and transported at 4°C to the microbiology laboratory of UNAN-León. Samples were then frozen at −20°C and transported to Linköping University, Sweden, for further analysis. Prior to sample collection, temperature and pH were registered. To avoid carryover contamination, tubes containing the samples were not opened until ultracentrifugation.

### Sample clarification and virus concentration

Before processing, samples were thawed overnight at 4°C, and clarified by centrifugation at 3,500× *g* for 5 min at 4°C. A total of 38 ml of supernatant were transferred to a thin wall, Ultra-Clear™ ultracentrifugation tube (Beckman, 344058). Ultracentrifugation was carried out in the Beckman Coulter - Optima™ L-100XP preparative ultracentrifuge (Beckman Coulter, Inc, CA, USA) using a SW 32 Ti Rotor at 164,000 g for 2 hours. A total of 37.5 ml of the supernatant was discarded by pipetting and the pellet was re-suspended in 0.5 ml of supernatant and frozen at −20°C until viral RNA purification. Distilled water was used as negative control in every round of centrifugation.

### RNA extraction

Viral RNA was extracted from 140 µl of the diluted pellet, following the manufacturer's instructions using a QIAamp Viral RNA Mini Kit (QIAGEN, Hilden, Germany). A total of 60 µl of viral RNA was collected and stored at −70°C until reverse transcription (RT).

### Reverse transcription

RT was essentially carried out as described previously [9], [21]. Briefly, 28 µl of RNA was mixed with 50 pmol of random hexadeoxynucleotides [pd(N)6] (GE Healthcare Bio-Sciences AB, Uppsala, Sweden), denatured at 97°C for 5 min, and quickly chilled on ice for 2 min, followed by addition of one RT-PCR bead (GE Healthcare Bio-Sciences AB) and RNase-free water to a final volume of 50 µl. The RT reaction was carried out for 30 min at 42°C to synthesize the cDNA used for screening and quantification of RV and NoV.

### Detection and quantification of RV and NoV

RV was detected, subgrouped and quantified using a real-time PCR assay [5]. The assay can subgroup RV strains based on subgroup specific fluorogenic primers targeting the VP6-gene, and semi-quantify using external plasmid standards.

NoV detection and quantification were performed with a real-time PCR targeting the ORF1-ORF2 junction, as described by Nordgren and co-workers [22]. This real-time PCR assay can semi-quantify and distinguish between NoVs GI and GII [22].

### Determination of NoV and RV concentrations and reduction

By using a standard curve in each real-time PCR assay as described elsewhere [5], [22], the number of viral genomes per real-time PCR reaction was determined, thus allowing us to estimate the number of both NoV and RV particles per liter of wastewater. The lowest detection level of NoV and RV in the real-time PCR assay is theoretically 1 gene per PCR reaction which corresponds to 2.5×10^3^ genome equivalents (g.e.) per liter (l^−1^) of H_2_O using the methodology described in this paper. Furthermore, quantification of concentrations lower than 2.5×10^4^ g.e. l^−1^ H_2_O (10 genes per PCR reaction) is less accurate due to less linearity of the real-time PCR standard curve close to the detection limit. The reduction of virus in the wastewater treatment plant was determined as the log_10_ transformation of the quotient of amount of virus before treatment, and the amount of virus after treatment. Due to the limited detection of RV in wastewater, the reduction was only determined for NoV. When NoV was below the detection limit in the outgoing water, a hypothetical value of 1.25×10^3^ g.e. l^−1^ H_2_O (which corresponds to approximately half of the detection limit) was given for measurement of average reduction levels [12], [23].

### RV VP7 genotyping and sequencing

A PCR mix was prepared by mixing 2.5 µl of cDNA, 4 pmol of each consensus primer (VP7-F and VP7-R) [24], 20.5 µl of RNase-free water and a PuReTaq Ready-To-Go PCR bead (GE Healthcare Bio-Sciences AB). PCR was performed at 94°C for 2 min, followed by 35 cycles of 94°C for 1 min, 50°C for 1 min, and 72°C for 1 min, with a final extension of 72°C for 7 min. The VP7 PCR-product was stored at −20°C until VP7 genotyping. A PCR mix was prepared by mixing 1 µl of VP7 PCR product, 4 pmol of each G genotype-specific and consensus primers (G1, G2, G3, G4, G8, G9 and VP7-R) [24], 23 µl of RNase-free water and a PuReTaq Ready-To-Go PCR bead (GE Healthcare Bio-Sciences AB). PCR was performed at 94°C for 4 min, followed by 30 cycles of 94°C for 1 min, 42°C for 2 min, and 72°C for 1 min, with a final extension of 72°C for 7 min. The PCR products were analyzed using 2% agarose gel and ethidium bromide staining, VP7 genotypes were assigned as described elsewhere [24].

The VP7 gene from one sample, containing sufficient RV concentration to enable an amplification of the VP7-gene, was sequenced using BigDye chemistry at Macrogen Inc. (Seoul, South Korea), with the primers VP7-F and VP7-R [24] as sequencing primers.

### PCR amplification and cloning of the NoV N-terminal and shell (NS) region

PCR amplification of the NS region was performed using the forward primer [22] and reverse primer [25] as described elsewhere [9]. The NoV NS capsid fragment was cloned into pJET1.2 vector and transformed into *E.coli* cells using CloneJet™ PCR Cloning and TransformAid™ Bacterial Transformation kits (Fermentas, St. Leon-Rot, Germany) according to the manufacturer's instructions. Ten separate colonies from each transformation reaction were screened by PCR for the correct gene insert. After overnight incubation of positive colonies, plasmid DNA was extracted and purified using GeneJET™ Plasmid Miniprep Kit (Fermentas, St. Leon-Rot, Germany) according to the manufacturer's instructions. Nucleotide sequencing was performed by Macrogen Inc. (Seoul, South Korea), using the BigDye chemistry with pJET1.2 forward and reverse sequencing primers.

### Genotyping of NoV by sequence analysis of the NS region

Sequence alignment of the Nicaraguan strains and reference NoV genotypes representing known genotypes as well as GII.4 variants, was performed by using the ClustalW algorithm, version 2, at the European Bioinformatics Institute server (EMBL-EBI). Phylogenetic analysis was performed using the MEGA 5.0 software package, and the tree was constructed using the neighbor-joining and Kimura two parameter methods. Significance of the relationship was obtained by bootstrap re-sampling analysis (1,000 replications).

### Nucleotide accession numbers

The nt sequences as used for phylogenetic analysis (nt position 5085 to 5366; Ref acc.no X86557) of the norovirus NS region have been deposited in GenBank under accession numbers JN102111–JN102131.

### Statistical analysis

Statistical analysis was performed using SPSS software (version 19, SPSS, Chicago, IL). Differences in RV and NoV prevalence in sewage water were determined by the use chi-square tests or Fisher's exact test. P-values of <0.05 were considered statistically significant.

## Results

### Low prevalence of RV and high prevalence of NoV in hospital and community wastewater

All wastewater samples from July 2007 to July 2008 were tested for RV and NoV using sensitive real time-PCR methods [5], [22] and the results demonstrated RV and NoV in 9 (11%) and 37 (44%) of the 84 sewage samples, respectively. Of the 9 RV-positive samples, 7 were also positive for NoV. The prevalence of RV in samples collected at the hospital wastewater, WWTP-inflow and WWTP-outflow were 7% (2/28), 21% (6/28) and 4% (1/28), respectively. In contrast, a higher NoV prevalence was observed at all sampling points, with 46% (13/28) in the hospital water, 54% (15/28) at the WWTP-inflow and 32% (9/28) at the WWTP-outflow (p<0.05; for all sampling points) (Table 1).

**Table 1:** Temporal distribution of rotavirus and norovirus in wastewater samples post RV5 introduction in León, Nicaragua, 2007–2008.

Month of Collection	No. of samples	No. of RV-positive/tested samples			No. of NoV-positive/tested samples		
		Hospital	Inflow WWTP	Outflow WWTP	Hospital	Inflow WWTP	Outflow WWTP
Jul 07	12	0/4	**2/4**	**1/4**	**3/4**	**4/4**	**4/4**
Aug 07	15	0/5	**2/5**	0/5	**3/5**	**3/5**	**1/5**
Sept 07	12	**2/4**	**1/4**	0/4	**2/4**	**2/4**	**2/4**
Oct 07	12	0/4	0/4	0/4	0/4	0/4	0/4
Nov 07	9	0/3	0/3	0/3	0/3	**1/3**	0/3
Dec 07	6	0/2	0/2	0/2	**2/2**	**1/2**	**1/2**
Feb 08	3	0/1	**1/1**	0/1	0/1	**1/1**	**1/1**
Mar 08	3	0/1	0/1	0/1	0/1	**1/1**	0/1
Apr 08	3	0/1	0/1	0/1	0/1	0/1	0/1
May 08	3	0/1	0/1	0/1	**1/1**	0/1	0/1
Jun 08	3	0/1	0/1	0/1	**1/1**	**1/1**	0/1
Jul 08	3	0/1	0/1	0/1	**1/1**	**1/1**	0/1
**All months**	**84**	**2/28 (7.1%)**	**6/28 (21.4%)**	**1/28 (3.6%)**	**13/28 (46.4%)**	**15/28 (53.6%)**	**9/28 (32.1%)**

### Seasonality of RV and NoV in the wastewater samples

This epidemiological parameter was investigated in the samples collected at the inflow of the WWTP, as they best represent the viruses circulating in the community. RV-positive samples were observed in July (2/4), August (2/4) and September (1/4) of 2007, and disappeared in the following months of surveillance, except in February (1/1) of 2008 (Table 1). An interesting observation was that RV seemed to circulate in the community a few weeks in July and August before its appearance in the hospital sewage water in September 2007 (Table 1). While RV circulated in very short time frames, NoV circulated year around, and only in the months of October of 2007 and April–May 2008 was it absent in wastewater (Table 1). NoV seemed to exhibit two periods of high concentration in incoming wastewater, during the rainy season (July to September), and during the winter season (November to March) while RV was mainly detected during the rainy season (July to September 2007) (Table 1).

### Higher concentrations of NoV compared to RV in hospital and community wastewater samples

**Figure 1:**
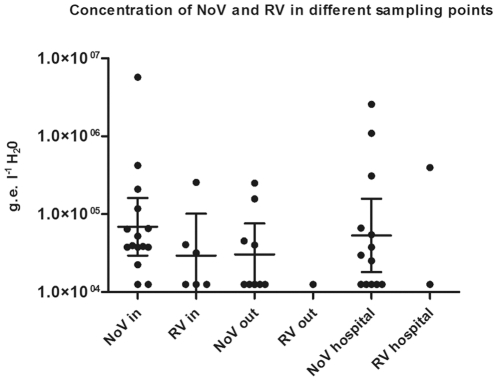
Concentrations of NoV and RV in different sampling points. The horizontal lines represent geometric mean with 95% confidence interval.

To investigate if RV and NoV quantities in the sewage were related to frequency, time and place of collection, we measured viral concentrations for NoV and RV in all sampling and time points. The frequency of RVs was low as compared to NoVs, which was also reflected in the quantification data (Fig. 1). The concentration of NoV ranged between 1.3×10^4^ to 5.7×10^6^ g.e. l^−1^ H_2_O. The highest concentration of NoV was found in hospital sewage in September 2007 (2.6×10^6^ g.e. l^−1^ H_2_O) and in the inflow to the WWTP in December 2007 (5.7×10^6^ g.e. l^−1^ H_2_O). The concentration of RV ranged between 1.3×10^4^ and 4.0×10^5^ g.e. l^−1^ H_2_O. The highest concentrations of RV were found in the inflow of the WWTP in September 2007 (2.6×10^5^ g.e. l^−1^ H_2_O) and in hospital sewage in September 2007 (4.0×10^5^ g.e. l^−1^ H_2_O) (Fig. 1). We furthermore measured the reduction level of NoV in the WWTP at all time-points where NoV was detected in incoming wastewater (n = 15), and observed that NoVs were reduced on average 1.15±0.29SE log_10_ units in the treatment process.

### The majority of all RVs detected belonged to subgroup I

**Figure 2:**
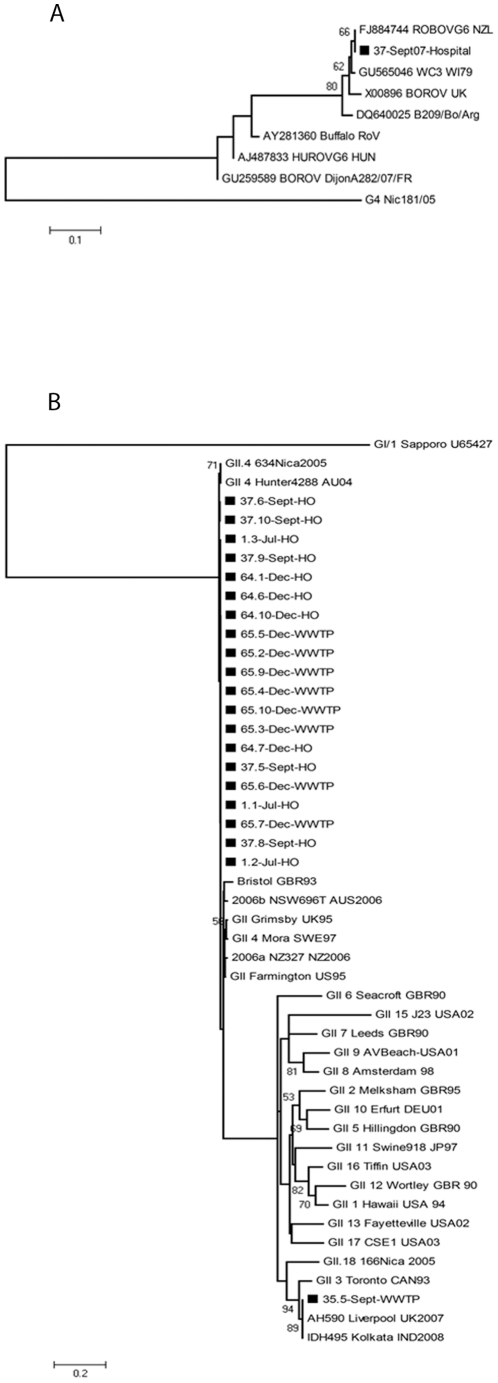
Phylogenetic analysis of (A) a RV VP7-gene fragment (165 bp, nt 637 to 801, ref acc.no. M12394) isolated from sewage water collected at the local hospital of León, Nicaragua in September 2007. (B) Partial capsid NS region (282 bp; 5085 to 5366; ref acc.no. X86557) of NoV GII cDNA (n = 21) isolated from sewage water collected at the local hospital and the WWTP of León, Nicaragua in 2007. Both trees were constructed based on the Kimura two-parameter and neighbor-joining methods by use of MEGA 5.0 software. Bootstrap values are shown at the branch nodes (values of <50% are not shown). RV G4Nic181/05 and Sapporo virus GI/1 (ref. acc. no. U65427) were used as outgroups, respectively. For Nicaraguan strains isolated in this study, the number of the sample and clone is given, followed by month of sample collection and site of collection (HO: Hospital; WWTP: Wastewater Treatment Plant).

Of the 9 RV-positive samples, 6 (67%) belonged to SGI, and 3 (33%) belonged to SGII. All RV-positive samples found in the hospital wastewater (n = 2) belonged to subgroup SGI; and were collected in September 2007. The two RV subgroups were not simultaneously observed in any samples investigated. We sequenced the VP7 gene of one SGI RV-positive (37-Sept07-Hospital) which had relatively high viral load. This sample was previously negative for the following G-types; G1, G2, G3, G4, G9, G8 and G10, using the nested multiplex PCR assay (data not shown). BLAST analysis of a short 165 nt segment of the VP7-gene revealed high nucleotide homology (99%) to bovine G6 strains isolated in New Zealand (RotaBovG6-NZ), China (CHLY) and India (B-85). Moreover, the nucleotide sequence was also homologous (98% nt similarity) to the human-bovine reassortant vaccine strain (P1 WC3×WI79 [WI79-4]) (Fig. 2A).

We furthermore detected G2 and G4 VP7-specificities, as determined by the nested multiplex PCR assay, in the inflow to the WWTP in July (n = 1) and September 2007 (n = 1), respectively. The VP6 subgroup determination in both samples revealed SGI and SGII specificities, respectively.

### The majority of NoVs belonged to genotype GII.4

Of the 37 NoV-positive samples, 28 (76%) were GII strains and 2 (5%) were GI strains, while both NoV genogroups were detected in 7 (19%) of the samples. To study NoV diversity, NoV-positive sewage samples with high viral load were selected for cloning and nucleotide sequencing, and for a subset of these (n = 5) collected in July, September and December of 2007 we obtained clones. In total 21 clones containing a segment (nt position 5085 to 5366) of the NS region of NoV were sequenced, 20 (95%) of these were highly homologous (99% nt similarity) to GII.4 NoV strains. One NoV clone, observed in the inflow of wastewater in September 2007, was highly homologous (100% nt identity) to GII.3 NoV isolated from children with community acquired diarrhea in October 2007 in UK, and to a GII.3 NoV strain isolated from a 30 year old male from Kolkata, India in 2008 (Fig. 2B).

## Discussion

In this study we investigated the prevalence of NoV and RV in hospital and municipal wastewaters in order to understand the transmission of these viruses at community and hospital level following implementation of RV vaccination in Nicaragua.

The major novel finding was the low prevalence of RV, further emphasized by the high prevalence of NoVs in both hospital and municipal wastewater. Several studies have reported higher frequencies of RV as compared to NoV in wastewater. Research groups from China, Tunisia and Germany described detection rates of RV ranging from 32% to 90% and NoV from 3% to 31% [26], [27], [28]. In hospital wastewater, Prado and coworkers observed RV in 95% and NoV GII in 28.5% of the samples collected between 2005 and 2008 in Rio de Janeiro, Brazil. [29]. In the current study mean RV prevalence in wastewater from different sources was only 11%, whereas, it was 44% for NoV (p<0.05). Moreover, in hospital wastewater, NoV was detected in 46.4%, whereas RV was only detected in 7.1% of the samples. This suggests a low frequency of severe hospital-requiring RV infections in the community as recently reported [30]. Furthermore, differences were not only observed in virus prevalence, but also in virus concentration; in average RV concentrations were lower as compared to NoV concentrations, both in hospital and in community wastewater. These observations suggest a high NoV transmission at population level in León, Nicaragua, during the time frame investigated, which is supported by the large burden of diarrhea that still persists in León after RV5 introduction [20].

Taken together, these observations strongly suggest that the implementation of RV5 in the EPI reduced the transmission of RV in León, Nicaragua both at community and hospital level. A recent report further supports this suggestion indicating that the RV immunization program in León is effective in reducing diarrhea episodes during the RV season [20]. In earlier studies from Nicaragua, the dry months (February–April) are usually associated with high rates of RV-induced diarrhea [21], [31]. In this study, however, most RVs were detected in wastewater during the rainy season (July–September) indicating a shift in the epidemiological pattern for RV. A similar shift has also been observed in a study from United States, reporting a delay of 15 and 8 weeks in the onset and peak of the RV season in 2007 and 2008 respectively, compared to the pre-vaccine periods 2000–2006 [32]. However, the differences in detection rates could also be partially attributed to the study design. Sample collection was not uniform throughout the year; samples were collected weekly at the WWTP during the rainy season and until Christmas holidays (July–December 2007), whereas samples were collected monthly during the remainder of the study period.

The real-time PCR assays used for detection and quantification of these viruses are based on the same chemistry and have similar efficiencies [5], [22]; therefore the difference in observed prevalence should not be due to differences in the detection methods, neither due to the concentration method, as ultracentrifugation has been shown to be more efficient to recover RV from wastewater as compared to ultrafiltration-adsorption-elution method [33].

The NoV concentrations in incoming wastewater for treatment ranged from 1.3×10^4^ to 5.7×10^6^ g.e l^−1^ H_2_O. The NoVs were reduced on average 1.15±0.29SE log_10_ units in the WWTP of León, albeit with variations in reduction between different time points. Furthermore, NoV was found in 32.1% of the outgoing wastewater samples from the treatment plant, demonstrating high transmission of NoV into the water environment even after the treatment process, suggesting that a disinfection step is needed. RV, however, was only detected in one outgoing wastewater sample (3.6%) (Table 1). The low reduction of NoV in the WWTP with subsequent high transmission into the environment is of concern, due to the environmental stability of the virus and its association with food and waterborne outbreaks [34], [35].

We characterized RV and NoV by sequencing. The RV concentration in wastewater was low, thus only one partial sequence of the VP7 gene from a water sample where we detected RV SGI-specificity with the real-time PCR assay, designated “37-Sept07-Hospital”, detected in hospital wastewater in September 2007 was obtained. The sequencing data demonstrated relatedness with animal RVs, having a high nucleotide homology (99%) with bovine strains detected in New Zealand, China and India belonging to the G6 genotype, which is the most common genotype found in calves elsewhere [36], [37]. The “37-Sept07-Hospital” sequence was also highly similar (98% nt homology) with the human-bovine reassortant vaccine strain (P1 WC3×WI79 [WI79-4]), which contains a VP7 protein with G6-specificity of bovine origin [38]. However, the sample containing this bovine-like RV strain was negative for the human G1, G2, G3, G4, G8, G9 and G10 genotypes. The RV5 contains five human-bovine reassortant strains, each with a different human VP7 (G1, G2, G3 or G4) or VP4 (P[8]) specificities that could be detected by RT-PCR [39]. Virus excretion from children vaccinated with RV5 is seldom reported [40], but can occur [41]. Since this sample was isolated from the hospital wastewater, it is likely that it contains a bovine-like RV from symptomatic children. Previously, a zoonotic bovine RV strain with G8 specificity has been identified in a diarrheic child during the monitoring of RV vaccine program in Nicaragua in 2008 [42]. However, it cannot be ruled out or confirmed that it was the vaccine strain which was detected in this study, as more sequence data will be needed to elucidate this.

The majority (6/9) of RV-positive wastewater samples were of SGI specificity, which is generally associated with human rotavirus G2P[4] [4]. In the current study RV G2 was observed in one wastewater sample, therefore we can speculate but not prove that RV of G2P[4] specificity were circulating in León during the time frame investigated, but at very low frequency. This speculation is strengthened by the fact that G2P[4] represented 88% of the RVs identified in a hospital surveillance carried out in the capital city of Nicaragua (Managua) between 2007 and 2008 [43]. The circulation of RV with P-types not included in the RV5 vaccine, such as P[4] and P[6] might be an expected phenomena after national vaccine implementation [43]. Previous studies from Nicaragua investigating RV genotypes in 2001–2003, 2005 and 2006 [21], [31], [44], found G2P[4] to be the dominating genotype in 2001, but since then circulating at low frequencies. The observed high prevalence of G2P[4] in Nicaragua 2007 and 2008 is likely due to naturally occurring shifts, rather than selection by RV5 introduction, since a very similar re-emergence of G2P[4] strains has been observed in other South- and Central-American countries, where no nationwide vaccine has yet been introduced [45].

Moreover, it cannot be ruled out that some of the SGI RV strains observed in wastewater were derived from animals, since SGI RVs are more common in animals than in humans [4], [46]. In the city of León, cattle and other domesticated animals are common and the sewage system also collects dump from these animals.

We observed a low frequency of GI NoVs (5%), and the few samples positive for this genogroup had low concentrations, which made it difficult to further characterize. There are reports from Japan, France and Sweden showing higher frequency of GI NoVs during some months, indicating a high transmission of these viruses in the community [12], [47], [48]. NoV GI has often been associated with food and waterborne outbreaks [49], [50], [51]. In this study, NoV GII was the most prevalent genogroup throughout the time-period investigated. Samples containing high NoV GII concentrations (>1×10^5^ g.e l^−1^ H_2_O) from different sampling and time points were selected for cloning and sequencing. We characterized NoV GII strains from hospital sewage in July, September and December 2007, as well as the inflow to the WWTP in September and December 2007. All NoV clones from the hospital wastewater were genotyped as GII.4 (Fig. 2B). NoV GII.4 has previously been the most common NoV strains detected in pediatric diarrhea in Nicaragua, and are ubiquitous worldwide [9], [52]. As described elsewhere [52], the GII.4 Hunter strain was globally replaced by GII.4 2006a/b in early 2006. However, the short segment of the NS region we sequenced in this study is not sufficient to accurately determine the exact GII.4 variant observed. The fact that all clones from the hospital wastewater were genotyped as NoV GII.4 reflects the importance of this genotype in NoV-associated diarrhea in Nicaragua. We found one NoV GII.3 strain in September 2007 in the inflow of the WWTP. The fact that a NoV GII.3 strain was only observed in the municipal wastewater and not in the hospital wastewater might suggest that NoV GII.3 was circulating at community level causing less severe or asymptomatic infections.

The detection of NoV GII.4 strains in July, September and December demonstrates a stable circulation of NoV GII.4 in Nicaragua after RV vaccine introduction. Usually, NoV GII.4 variants are associated with seasonal epidemics and a given variant predominate for a period of time until replaced by a new variant [53]. The presence of one particular NoV variant throughout the year in the current study is interesting, as higher NoV diversity have been observed in water samples from other studies [47], [54]. However, the differences could be attributed to temporal fluctuations of NoV strains in the community; no clinical data regarding NoV genotypes is available for the same time-period investigated. In an earlier clinical report from Nicaragua, a high diversity of NoV genotypes was found in April to June in children with diarrhea, whereafter GII.4 strains dominated [9]. Unfortunately, in the current study no wastewater samples collected between April and June had sufficient viral concentration to enable NoV genotyping. Whether a change of dynamics of NoV circulation occurs due to the introduction of RV vaccine remains to be elucidated

To conclude, this paper demonstrated low prevalence of RV and high prevalence of NoV in wastewater in León Nicaragua during the post RV vaccination period. The results suggest that implementation of the RV5 vaccine has reduced RV circulating in the community. Determination of RV and NoV in wastewaters may function as a surrogate marker for their prevalence in the society, and thus be used as a tool for monitoring vaccine efficacy.
